# Adaptation of a mouse Doppler echocardiograph system for assessing cardiac function and thermal performance in a juvenile salmonid

**DOI:** 10.1093/conphys/coab070

**Published:** 2021-09-03

**Authors:** Carlie A Muir, Bryan D Neff, Sashko Damjanovski

**Affiliations:** Department of Biology, Western University, London, Ontario, Canada N6A 5B7

**Keywords:** thermal performance, Doppler echocardiography, cardiorespiratory performance, cardiac function, Atlantic salmon

## Abstract

Measures of cardiac performance are pertinent to the study of thermal physiology and exercise in teleosts, particularly as they pertain to migration success. Increased heart rate, stroke volume and cardiac output have previously been linked to improved swimming performance and increased upper thermal tolerance in anadromous salmonids. To assess thermal performance in fishes, it has become commonplace to measure the response of maximum heart rate to warming using electrocardiograms. However, electrocardiograms do not provide insight into the hemodynamic characteristics of heart function that can impact whole-animal performance. Doppler echocardiography is a popular tool used to examine live animal processes, including real-time cardiac function. This method allows for nonsurgical measurements of blood flow velocity through the heart and has been used to detect abnormalities in cardiovascular function, particularly in mammals. Here, we show how a mouse Doppler echocardiograph system can be adapted for use in a juvenile salmonid over a range of temperatures and timeframes. Using this compact, noninvasive system, we measured maximum heart rate, atrioventricular (AV) blood flow velocity, the early flow-atrial flow ratio and stroke distance in juvenile Atlantic salmon (*Salmo salar*) during acute warming. Using histologically determined measures of AV valve area, we show how stroke distance measurements obtained with this system can be used to calculate ventricular inflow volume and approximate cardiac output. Further, we show how this Doppler system can be used to determine cardiorespiratory thresholds for thermal performance, which are increasingly being used to predict the consequences that warming water temperatures will have on migratory fishes.

## Teaser

Small rodent pulse-wave Doppler echocardiograph systems can be adapted for use in juvenile salmonids to measure metrics of cardiac function relevant to thermal performance, including maximum heart rate, ventricular filling volume, Arrhenius breakpoint temperature and arrhythmia temperature.

## Introduction

Cardiac performance, the capacity of the heart to deliver oxygenated blood to tissues, is the cornerstone of aerobic exercise. In anadromous salmonids, thermal limitations to cardiac performance are known to impact migration success (Farrell *et al.*, 2008). Eliason *et al.* (2011) showed that cardiorespiratory performance and morphology were locally adapted to both migration difficulty and historic river temperatures across populations of Fraser River sockeye salmon (*Oncorhynchus nerka*). Similarly, Gradil *et al.* (2016) demonstrated that in captive-bred Atlantic salmon (*Salmo salar*), juvenile cardiac performance was adapted to the thermal and hydrological conditions of their ancestral natal streams. Studies have identified juvenile survival during freshwater stages as a critical target in the conservation of threatened salmonid populations (Kareiva *et al.*, 2000; Zabel *et al.*, 2006), and elevated river temperatures have been associated with increased mortality in juvenile Chinook salmon (*Oncorhynchus tshawytscha*; Crozier and Zabel, 2006). Assessments of heart function and the thermal limits for cardiac performance are therefore critical in understanding the impacts of rising river temperatures on salmonids, towards conserving these ecologically and economically important species.

The proposed reliance of upper temperature tolerance on cardiac performance stems from the notion that in a variety of aquatic ectotherms, upper thermal limits are set by a mismatch between oxygen supply and demand (Brett, 1971; Portner and Knust, 2007). Aerobic scope is the difference between an organism’s routine and maximum oxygen consumption and represents the capacity to perform activities beyond meeting basal metabolic requirements (Fry, 1947). Fry curves, which describe the relationship between aerobic scope and temperature, demonstrate that aerobic scope is maximized at an optimum temperature (T_Opt_) and declines with further warming, until falling to zero at the organism’s upper critical temperature (T_Crit_). During warming, heart rate increases as the primary mechanism of supporting the heightened oxygen demands of tissues (Clark *et al.*, 2005; Farrell, 2009). However, at an inflection point known as the Arrhenius breakpoint temperature (T_AB_), the rate at which maximum heart rate (*f*_Hmax_) increases with temperature declines (Yeager and Ultsch, 1989; Casselman *et al.*, 2012). Above this T_AB_, oxygen availability may become limited for activities beyond meeting routine metabolic needs. As temperatures approach the upper limit of a fish’s thermal window, heart rate becomes irregular and cardiac function deteriorates, at what is known as the arrhythmia temperature, T_Arr_ (Clark *et al.*, 2008; Farrell, 2009; Casselman *et al.*, 2012). In Coho salmon (*Oncorhynchus kisutch*), it was demonstrated that these rate transition temperatures for *f*_Hmax_ (T_AB_ and T_Arr_) can be used to estimate the optimum temperature for aerobic scope (T_opt_) and the temperature at which aerobic scope collapses (T_crit_) (Casselman *et al.*, 2012; Anttila *et al.*, 2013a), respectively. This method developed by Casselman *et al.* (2012) involves pharmacologically stimulating maximum heart rate (*f*_Hmax_) in anaesthetized fish and recording the response of *f*_Hmax_ to warming as a surrogate measurement for aerobic scope. As generating a single Fry curve can take several weeks, determining thermal discontinuities in *f*_Hmax_ using electrocardiograms (ECGs) has been popularized as a high-throughput method of examining thermal performance of aerobic function in fish. This method has been applied in numerous salmonid species (Chen *et al.*, 2013, 2015; Anttila *et al.*, 2014; Muñoz *et al.*, 2015; Gradil *et al.*, 2016; Hansen *et al.*, 2017; Gilbert *et al.*, 2020), as well as goldfish (Ferreira *et al.*, 2014) and zebrafish (Sidhu *et al.*, 2014).

However, this method may not be appropriate in the study of marine species, as seawater hinders the ability of external electrodes to detect ECGs (Skeeles *et al.*, 2020). A growing number of studies have measured heart rate in fishes using more invasive methods, such as internal electrodes and implanted heart rate loggers (Clark *et al.*, 2009; Raby *et al.*, 2015; Ekström *et al.*, 2017; Prystay *et al.*, 2017; Brijs *et al.*, 2019; Safi *et al.*, 2019; Skeeles *et al.*, 2020). However, some studies employing internal heart rate loggers report successful data capture in only approximately half of test fish (Clark *et al.*, 2009; Skeeles *et al.*, 2020). This may be partly due to insufficient recovery time following implantation, as it has been recommended that fish are given at least 72 hours of post-surgical recovery time before heart rate measurements are taken (Brijs *et al.*, 2019). While such heart rate measurements can provide valuable insight into cardiorespiratory performance, this is just one component of cardiac output. Increased ventricle size and pumping capacity, which contribute to stroke volume, have also been linked to improved thermal tolerance and swimming performance (Franklin and Davie, 1992a; Claireaux *et al.*, 2005; Eliason *et al.*, 2011; Anttila *et al.*, 2013b). Thus, technologies that enable nonsurgical measures of heart rate, as well as hemodynamic parameters of cardiac function, are needed.

Echocardiography is a popular tool for assessing cardiac function, as it provides a noninvasive method for visualizing heart structures and measuring blood flow in real time. There are multiple modes of echocardiography [m-mode, b-mode and pulsed-wave (PW) Doppler], which use high-frequency sound waves to either produce images of intracardiac structures or measure hemodynamic characteristics. The PW Doppler mode can be used to measure the velocity of blood flow at valve openings based on the frequency shift of emitted soundwaves relative to returning echoes. Data are presented in real time as velocity spectrograph waveforms, which contain a number of important cardiac function parameters, such as heart rate, peak blood flow velocities and stroke distance. At the atrioventricular (AV) valve, these velocity waveforms display two distinct peaks: the early (E)-wave velocity, which represents passive filling of the ventricle, and the atrial (A)-wave velocity, which represents active atrial systole. The ratio of these two velocities, known as the E/A ratio, is a key indicator of diastolic function. The combined area underneath these waveforms represents the stroke distance, or distance travelled by the blood. When area of the AV valve is known, stroke distance can be used to determine ventricular inflow volume (Lee *et al.*, 2014). This ventricular inflow volume can subsequently be used as a proxy for stroke volume to approximate cardiac output, in conjunction with heart rate. In all, PW Doppler offers an opportunity to quickly and noninvasively measure additional parameters of cardiac function that can contribute to whole-animal performance.

**Figure 1:**
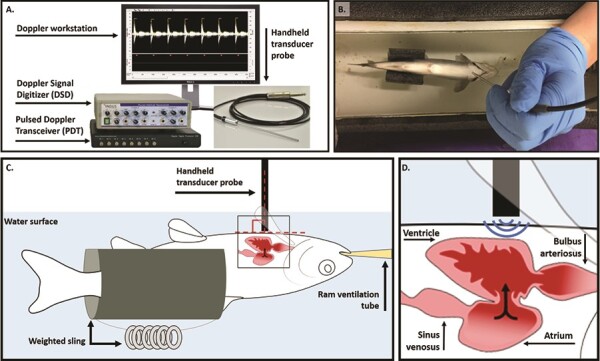
Indus DFV System and stabilization of a juvenile Atlantic salmon (*S. salar*) for echocardiograph measurements. (**A**) The Doppler echocardiograph system consists of a 20-MHz transducer probe (diameter, 2 mm), the PDT, the DSD and a PC computer equipped with the DSPW software. (**B**) The anaesthetized fish was placed ventral side up in the holding reservoir, submerged in water that was maintained at a set temperature by a recirculating water bath and RAM ventilated (ventral view). (**C**) A schematic of the fish (lateral view) supported by a weighted Styrofoam sling to ensure proper orientation of the fish for Doppler measurements. The transducer probe was positioned perpendicular to the ventral side of the fish posterior to the gills, such that (**D**) the probe was aligned with the AV valve of the heart, with blood flowing directly towards the probe.

To date, noninvasive Doppler imaging has been used in a variety of fishes to examine cardiac chamber morphology, detect cardiomyopathies and estimate cardiac output (Franklin and Davie, 1992b; Sande and Poppe, 1995; Lai *et al.*, 1998, 2004; Ho *et al.*, 2002; Claireaux *et al.*, 2005). Here, we show how a PW Doppler echocardiograph system, originally designed for use in mice, can be adapted to assess parameters of cardiac function in a juvenile salmonid (Atlantic salmon; *S. salar*, Linnaeus 1758). As echocardiography is a common technique in the medical sciences, single-mode systems designed for use in small mammalian models are readily available at relatively low costs. We chose to use the Indus Doppler Flow Velocity (DFV) System (Fig. 1A; Indus Instruments, USA), a compact system that was previously utilized with zebrafish in a myocardial infarction study by Benslimane *et al.* (2019). Benslimane *et al*. (2019) stabilized the test fish using a water-soaked sponge, which allowed for only a 5-minute measurement window before anaesthesia wore off and the fish was returned to a recovery tank. While this methodology is commonplace for echocardiography in zebrafish (Lee *et al.*, 2014; Kang *et al.*, 2015; Wang *et al.*, 2017), the lack of maintenance anaesthetics and temperature control limit its usefulness in longer physiological experiments. We show how this system can be used in conjunction with a recirculating, temperature-controlled water bath to obtain hemodynamic measurements of cardiac function during acute warming and assess the thermal performance of *f*_Hmax_ in anaesthetized fish. Further, we show how stroke distance measurements obtained using this system can be used in conjunction with measures of AV valve area to determine a proxy for stroke volume and subsequently, extrapolated cardiac output. In all, this method offers a compact, nonsurgical, high-throughput screening tool for assessing cardiorespiratory thresholds for thermal performance in fish, concurrently with additional parameters of heart function that may contribute to differences in whole-animal performance.

## Materials and methods

### Study population

All experiments were carried out according to Western University Animal Care protocol 2018-084. The Atlantic salmon used in this study descended from the LaHave River population (Nova Scotia, Canada; 44.4°N, 64.5°W) and were obtained as fertilized eggs from the OMNRF (Ontario Ministry of Natural Resources and Forestry) Normandale Research Facility (Vittoria, Ontario) in the Fall of 2017. Twenty-four hours after fertilization, eggs were transported to a hatchery facility at the University of Western Ontario and housed in egg trays at 7 ± 0.5°C. Once the endogenous yolk sacs of the fish were nearly absorbed, ~4 months post-fertilization, fry were transferred to 40-l Rubbermaid bins and the rearing temperature was raised to 11 ± 0.5°C. Increasing the temperature at this stage mimics seasonal temperature variation and aides in transitioning the salmon to exogenous feeding. Free-swimming fish were provided with pelleted feed *ad libitum* throughout the rearing period (EWOS Commercial Feeds, Bergen, Norway). Atlantic salmon were reared under these conditions for ~1.5 years post-hatching before Doppler echocardiography measures were performed during the parr stage.

### Stabilization of test fish for echocardiography

Juvenile Atlantic salmon (*N* = 8) were anaesthetized in water containing 100 mg l^−1^ of MS-222 buffered with 200 mg l^−1^ sodium bicarbonate, then weighed for body mass (10–25 ± 0.1 g). Once anaesthetized, the test fish was placed ventral side up in a purpose-made holding reservoir (Fig. 1B) and maintained at its rearing temperature (11°C) with recirculating water from a temperature-controlled water bath (VWR, Edmonton, Alberta, Canada). The holding reservoir (originally described in Gradil *et al.* 2016) was constructed from a short segment of PVC pipe (diameter, 4.5-inch) cut lengthwise and capped at both ends to form a semicircular holding trough (Fig. 1B). Individuals were maintained ventral side up in a weighted Styrofoam sling within the holding reservoir to ensure correct orientation of the fish for PW Doppler measurements (Fig. 1C). While in the holding reservoir, fish were ram ventilated with oxygenated water from the recirculating water bath, containing a maintenance dose of anaesthetic (75 mg l^−1^ of MS-222 buffered with 150 mg l^−1^ sodium bicarbonate). An additional inflow to the holding reservoir ensured the circulation of heated water (combined flow rate of 1.5 l min^−1^). This ram ventilation and temperature stabilization allows for measurements over an extended period of time, if desired. Fish were given a 15-minute stabilization period in the holding apparatus before DFV spectrograms were recorded. Reservoir water temperature was continually measured with a digital thermometer (Omega, St-Eustache, Quebec, Canada) to confirm the fish’s environmental temperature corresponded to the set temperature of the water bath.

**Figure 2:**
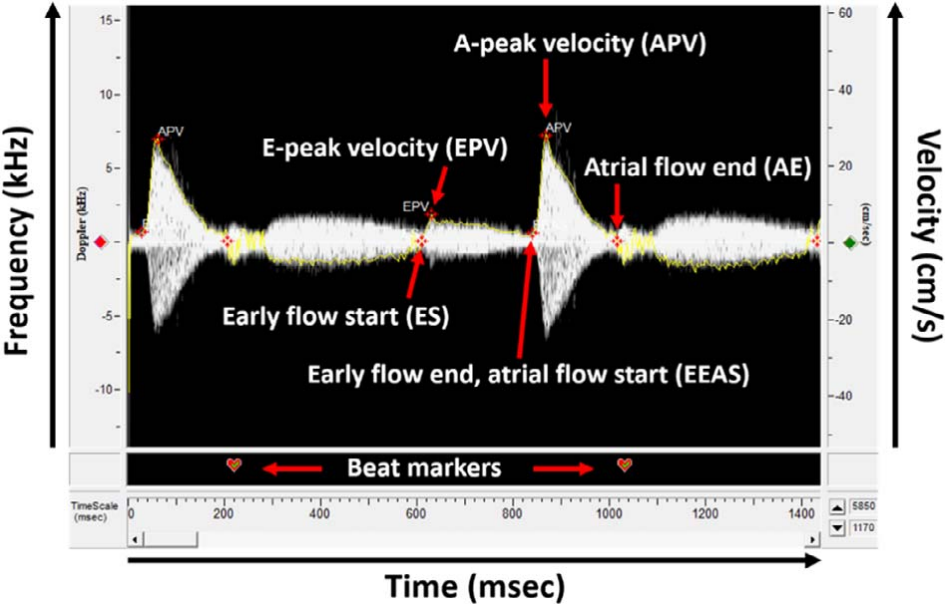
Doppler spectrograph analysed for parameters of AV blood flow. The ES marker represents the beginning of blood flow through the AV valve and is placed at the point where velocity increases from zero at the start of the E-wave. The EPV marker is placed at the maximum velocity of the E-wave. The EEAS marker is placed at the point where velocity begins to increase again following the E-wave, at the start of the A-wave as the atrium contracts. The APV marker is placed at the maximum velocity of the A-wave, which also represents overall peak blood flow through the AV valve. Finally, the AE marker is placed at the point where velocity returns to zero and represents the closing of the AV valve. Heart rate can be calculated by placing beat markers at the same landmark of each waveform.

### Blood flow measurements using the Indus DFV system

The 20-MHz transducer probe (diameter, 2 mm) was held perpendicular to the ventral side of the fish, directly posterior to the gills (Fig. 1). Based on known anatomy of the fish heart, this positions the probe such that blood moving through the AV valve flows directly towards it (Fig. 1D), and care was taken to ensure parallel alignment between the direction of the ultrasound beam and the direction of blood flow (Benslimane *et al.*, 2019). For each fish, probe adjustment was guided by previous observations that the highest measured velocity best approximates true blood flow velocity, since Doppler shift is maximized when insonation angle of the ultrasound beam approaches zero (Wang *et al.*, 2017). The small footprint of the DFV system probe (10-fold smaller than dual-mode clinical probes) eases parallel alignment with blood flow and it was previously shown that measurement error is below 1.5% when insonation angle is within 10° of the direction of blood flow (Reddy *et al.*, 2009). While previous applications made use of the system’s fixed probe mount, we found that movement of the fish relative to the mounted probe (due to water flow through the holding trough) led to inconsistencies in signal acquisition. These inconsistencies were reduced by holding the fish and probe in place and checking to ensure maximum signal velocity between serial measurements. Signals received by the Pulsed Doppler Transceiver (PDT) were digitized by the Doppler Signal Digitizer (DSD) and displayed on the Doppler Signal Processing Workstation (DSPW) as continuous, real-time grayscale DFV spectrograph waveforms (Fig. 1A).

### Analysis of velocity spectrograph waveforms for cardiac function metrics

Spectrograms were processed in the system’s DSPW software to calculate metrics of cardiac function. Technical specifications are provided in Supplemental Figure 1. Spectrograph waveforms were analysed for parameters of AV blood flow using the ‘Mitral Inflow’ mode in the software’s ‘Analysis Control Window’ (Supplementary Fig. 1). Heart rate (beats min^−1^) was calculated by identifying the number of peaks in a known time duration using the software’s ‘Beat Editor’. Using the ‘Marker Editor’, markers were placed at the ‘Early Flow Start’ (ES), ‘Early Flow Peak Velocity’ (EPV), ‘Early Flow End Atrial Flow Start’ (EEAS), ‘Atrial Flow Peak Velocity’ (APV) and ‘Atrial Flow End’ (AE) of each beat, as shown in Fig. 2. The software’s ‘Envelope Editor’ automatically traced the edges of each spectrogram with a yellow line, which is used to calculate stroke distance and aided in the placement of markers (Fig. 2). Based on these markers, the software calculated A-peak velocity, A-stroke distance, E-peak velocity, E-stroke distance and E-A peak velocity ratio. For each metric, the software averaged the values across selected beats (*n* = 10) and reported results as mean ± SD in the ‘Measurement Results’ tab. A-stroke distance and E-stroke distance represent the area under the A- and E-peaks, respectively. These values for area under the A- and E-peaks can be totalled to obtain the total stroke distance perbeat.

**Figure 3:**
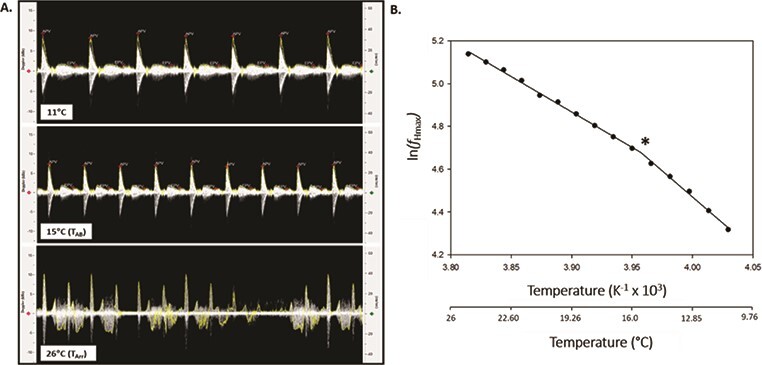
Determining cardiorespiratory thresholds for thermal performance using Doppler spectrographs. (**A**) Doppler spectrographs recorded at the acclimation temperature (11°C), Arrhenius breakpoint temperature (T_AB_; 15°C) and arrhythmia temperature (T_Arr_; 26°C) of a juvenile Atlantic salmon (*S. salar*). (**B**) Arrhenius breakpoint temperature (T_AB_) analysis of maximum heart rate (*ƒ*_Hmax_) for a juvenile Atlantic salmon exposed to an acute warming event following pharmacological stimulation of *ƒ*_Hmax_. The natural log of maximum heart rate (ln (*ƒ*_Hmax_)) was plotted against temperature, and the discontinuity in temperature-induced increases in *ƒ*_Hmax_, which represents T_AB_, was determined using a piecewise, two-segment linear regression. T_AB_ is denoted by *.

### Thermal performance of cardiac function

Once transferred to the holding reservoir, anaesthetized fish were maintained at their rearing temperature for a 15-minute stabilization period, after which DFV spectrograms were recorded to measure baseline heart rate and AV blood flow. According to Casselman *et al*. (2012), fish were pharmacologically stimulated to reach *f*_Hmax_ using intraperitoneal injections of 1.2 mg kg^−1^ atropine sulphate (Sigma-Aldrich, St. Louis, MO, USA) and 4 μg kg^−1^ isoproterenol (Sigma-Aldrich, St. Louis, MO, USA); both agents were dissolved in 0.9% NaCl. Atropine sulphate was used to block vagal tone, whereas isoproterenol was used to fully stimulate adrenergic β-receptors. Each injection was followed by a 15-minute equilibration period. A stepwise temperature increase was applied in 1°C increments every 6 minutes (Casselman *et al.*, 2012). After each 1°C increase, temperature and *f*_Hmax_ were allowed to stabilize briefly before spectrograph waveforms of the fish’s heartbeat were recorded (Fig. 3A). Spectrograms were manually saved in 30-second intervals (10 per temperature) for later analysis of *f*_Hmax_ and blood flow velocity. Once spectrograms displayed a shift from rhythmic to arrhythmic heartbeats (Fig. 3A), the fish was removed from the apparatus and euthanized by lethal overdose of MS-222.

T_AB_ was calculated for *f*_Hmax_ in SigmaPlot 13.0 (Systat Software, San Jose, CA, USA) as described by Munoz *et al.* (2015). Briefly, the natural logarithm of *f*_Hmax_ was plotted as a function of the inverse of temperature (K) to generate an Arrhenius plot for each fish (Fig. 3B). Using the software’s ‘Dynamic Fit Wizard’, a piecewise, two-segment linear equation was applied to fit a biphasic line to the data. The point at which the slope changed represents the Arrhenius breakpoint temperature, T_AB_ (Yeager and Ultsch, 1989) and corresponds to the temperature at which temperature-induced increases in *f*_Hmax_ shifted to a lower exponent (Fig. 3B). For each fish, we also identified the temperatures at which *f*_Hmax_, stroke distance and extrapolated cardiac output were maximized (denoted as T_peak*f*H_, T_peakSD_ and T_peakCO_, respectively). Cardiac arrhythmia temperature, T_Arr_, was identified as the temperature at which arrhythmias occurred in the spectrograph waveforms (Fig. 3A).

**Figure 4:**
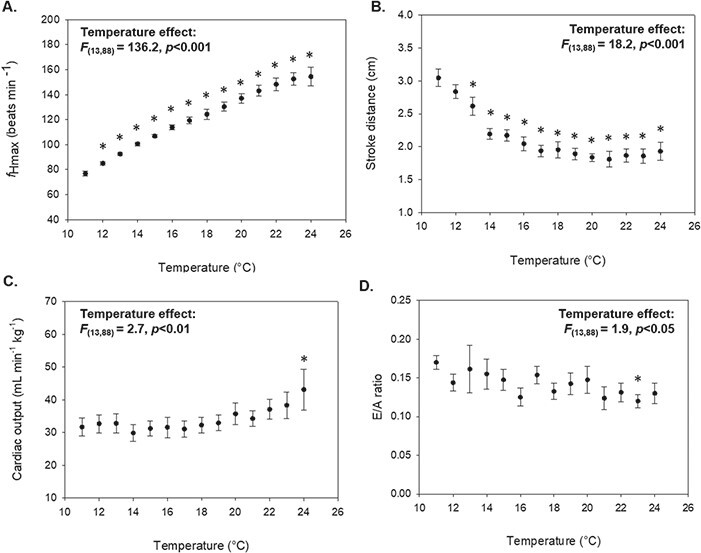
Influence of acute warming on cardiac performance in juvenile Atlantic salmon (*S. salar*; *N* = 8). Data are presented as means ± SEM for (**A**) maximum heart rate (*ƒ*_Hmax_), (**B**) stroke distance, (**C**) extrapolated cardiac output and (**D**) E/A ratio. Asterisks (*) denote statistically significant differences from the initial value at 11°C (*P* < 0.05).

### Histological measures of AV valve diameter and cardiac output calculations

Following DFV measures and euthanasia, fish were fixed in 10% neutral buffered formalin (VWR, Radnor, PA, USA) for subsequent histological sectioning. After 48 hours, the midsection of the fish was trimmed (anterior to the eyes and posterior to the pectoral fins) and returned to 10% neutral buffered formalin for an additional 72 hours. Following serial dehydration, the dissected specimen was stored in 70% ethanol and sent to the Robarts Molecular Pathology Facility (London, ON) for embedding and histological sectioning. Serial sections (thickness, 10 μm) were taken through the sagittal plane, traversing the heart and stained with haematoxylin and eosin. Sections were imaged using an Olympus SZX9 dissecting microscope equipped with an OPTIKA C-B5 camera and analysed in OPTIKA PROView (OPTIKA Srl, Ponteranica, Bergamo, Italy). The diameter of the AV valve opening was measured in serial sections to determine the maximum diameter, which was then used for subsequent analysis (Supplementary Fig. 2). In lieu of histological analysis, diameter of the AV valve can be measured directly in dissected hearts or estimated using scaling relationships based on body mass. Area of the AV valve was calculated using the formula $\pi {r}^2$, where *r* represents valve radius (Lee *et al.*, 2014). Ventricular inflow volume (cm^3^, or ml) was calculated at each temperature as the total stroke distance (cm) multiplied by the cross-sectional area of the AV valve (cm^2^; Lee *et al.*, 2014). In salmonids, changes in stroke volume are largely governed by changes in ventricular inflow volume (Frank–Starling mechanism; Franklin and Davie, 1992b; Keen *et al.*, 2017). Therefore, assuming that beat-to-beat variation in ejection fraction is low at a given temperature, ventricular inflow volume can be used as a proxy for stroke volume. Extrapolated cardiac output (ml min^−1^) was calculated by multiplying ventricular inflow volume (stroke distance multiplied by valve area) by heart rate at each temperature (Seymour *et al.*, 2020). For comparative purposes, ventricular inflow volume and extrapolated cardiac output are expressed per kg of body mass (ml kg^−1^ and ml min^−1^ kg^−1^, respectively).

### Statistical analyses

Statistical analyses were performed using SigmaPlot 13.0 (Systat Software, San Jose, CA, USA). Temperature effects on mean (± SEM) cardiac performance variables were assessed using a one-way repeated measures (temperature) ANOVA. Significant temperature effects were further explored by multiple comparisons (Holm–Sidak method) to identify points that were significantly different from the initial value at 11°C. Significance was considered at *P* < 0.05.

## Results

**Figure 5:**
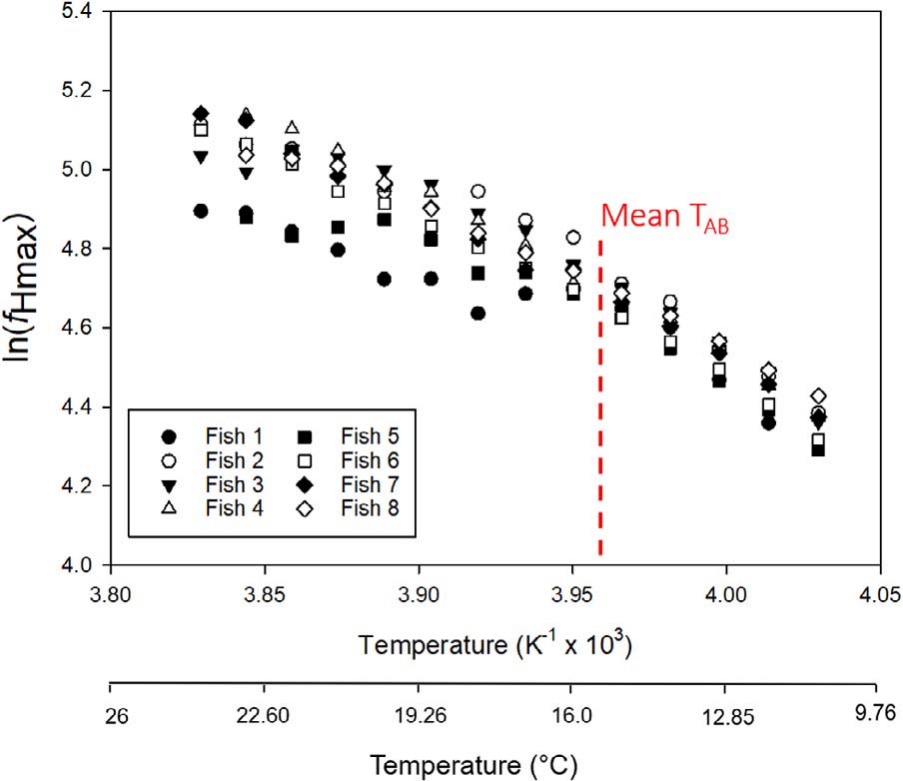
Modified Arrhenius plots of the natural log of maximum heart rate (ln (*ƒ*_Hmax_)) against temperature for individual Atlantic salmon (*S. salar*; *N* = 8) subjected to an acute warming event following pharmacological stimulation of *ƒ*_Hmax_. Red dashed line denotes the mean T_AB_ (15.7 ± 0.6°C).

Velocity signals were successfully obtained in all test fish across the range of experimental temperatures. Anecdotally, we have obtained velocity signals in juvenile salmon up to 50 g in size using this method. *f*_Hmax_ increased with temperature during acute warming (*F*_(13,88)_ = 136.2, *P* < 0.001; Fig. 4A), with peak *f*_Hmax_ occurring between 23°C and 25°C. The mean peak *f*_Hmax_ calculated from Doppler spectrographs was 157.0 ± 2.1 beats min^−1^ (Table 1), and the mean temperature at which peak *f*_Hmax_ occurred (T_peak*f*H_) was 23.8 ± 0.3°C (Table 2). The T_AB_ for *f*_Hmax_ was easily pinpointed in each of our test fish (Fig. 5), with a mean T_AB_ of 16.7 ± 0.7°C (Table 2). Onset of cardiac arrhythmias occurred between 24°C and 27°C and mean T_Arr_ was 25.0 ± 0.4°C (Table 2).

**Table 1:** Morphology and cardiac function measures (obtained using PW Doppler echocardiography) for juvenile Atlantic salmon (*S. salar*)

Measure	Mean
Body mass (g)	15.1 ± 1.8
AV valve area (cm^2^)	0.002 ± 0.3 x10^−4^
Peak *f*H_max_ (beats min^−1^)	157.0 ± 5.9
Peak APV (cm/s)	38.0 ± 2.1
Peak EPV (cm/s)	5.4 ± 0.4
Routine E/A ratio	0.17 ± 0.01
Peak stroke distance (cm)	3.1 ± 0.1
Peak inflow volume (ml kg^−1^)	0.43 ± 0.04
Peak cardiac output (ml min^−1^ kg^−1^)	42.2 ± 5.5

*Note*: fH_peak_, peak f_Hmax_.

Data presented as means ± SEM. *N* = 8.

**Table 2:** Thermal performance measures for juvenile Atlantic salmon (*S. salar*) obtained using the PW Doppler echocardiography

Measure	Mean
T_AB_ (°C)	15.7 ± 0.6
T_Arr_ (°C)	25.0 ± 0.4
T_peak*f*H_ (°C)	23.8 ± 0.3
T_peakSD_ (°C)	11.5 ± 0.3
T_peakCO_ (°C)	20.9 ± 1.5

*Note*: T_AB_, Arrhenius breakpoint temperature; T_Arr_, temperature at onset of cardiac arrhythmias; T_peakfH_, temperature for peak f_Hmax_; T_peakSD_, temperature for peak stroke distance; T_peakCO_, temperature for peak cardiac output.

Data presented as means ± SEM. N = 8.

In addition to measures of *f*_Hmax_, we took hemodynamic measurements of heart function during acute warming (Fig. 4). Stroke distance peaked at or near the fish’s routine temperature of 11°C (11–13°C) and subsequently declined with warming (*F*_(13,88)_ = 18.2, *P* < 0.001; Fig. 4B), resulting in a mean T_peakSD_ of 11.5 ± 0.3°C (Table 2). Measures of stroke distance were used to calculate ventricular inflow volume, in conjunction with measures of AV valve area (0.001–0.003 cm^2^, Table 1). Peak ventricular inflow volumes ranged from 0.26 to 0.62 ml kg^−1^ and mean peak inflow volume was 0.43 ± 0.04 ml kg^−1^. Acute warming had a significant effect on cardiac output (*F*_(13,88)_ = 2.7, *P* < 0.01; Fig. 4C); however, the temperature at which peak cardiac output occurred was highly variable, ranging from 13°C to 25°C (mean T_peakCO_ = 20.9 ± 1.5°C; Table 2). Peak cardiac output values ranged from 21.6 to 71.9 ml min^−1^ kg^−1^, with a mean value of 42.2 ± 5.5 ml min^−1^ kg^−1^. APV ranged from 31.1 to 47.0 cm/s (mean = 38.0 ± 2.1 cm/s), while EPV ranged from 5.0 to 7.6 cm/s (mean = 5.4 ± 0.4 cm/s). Mean E/A velocity ratio was 0.17 ± 0.01 (Table 1) at the routine temperature of 11°C and tended to decline as temperature increased (*F*_(13,88)_ = 1.9, *P* < 0.05; Fig. 4D).

## Discussion

For the first time in a salmonid, we used a small-rodent DFV system to assess multiple parameters of cardiac function across a range of experimental temperatures. Previous studies have demonstrated through co-registration of ECG and Doppler echocardiography that heart rates determined via frequency analysis of velocity waveforms are congruent with heart rate determinations from ECG (Lee *et al.*, 2014; Seymour *et al.*, 2020; Perez *et al.*, 2021). Measures of peak *f*_Hmax_ obtained here (mean of 157 ± 5.9 beats min^−1^; Table 1) are comparable to previously reported values for juvenile coho salmon (*O. kisutch*, 143 beats min^−1^; Casselman *et al.*, 2012), Chinook salmon (153 beats min^−1^; Muñoz *et al.*, 2015) and Atlantic salmon (149–176 beats min^−1^; Anttila *et al.*, 2014; Gradil *et al.*, 2016) obtained using the methodology of Casselman *et al.* (2012). Further, T_AB_ was successfully pinpointed in all test fish (mean of 16.7°C; Table 2), producing values that are similar to previous estimates in juvenile Atlantic salmon (14.7–17.0°C; Anttila *et al.*, 2014; Gradil *et al.*, 2016). These findings demonstrate that Doppler echocardiography can be used in place of ECG electrodes to reliably capture *f*_Hmax_ during acute warming and determine cardiorespiratory thresholds for thermal performance (Fig. 5).

In addition to measurements of *f*_Hmax_, we simultaneously measured a number of hemodynamic parameters of heart function that cannot be obtained using ECG, such as the E/A ratio (Table 2). The E/A velocity ratio is a commonly used metric for assessing diastolic function, or filling of the ventricle. Contrary to mammals, the peak velocity of the A-wave exceeds that of the E-wave in fish (Fig. 2), yielding routine E/A ratios less than 1 (Lee *et al.*, 2014). E/A ratios reported here (0.14–0.21 at 11°C) fell within the range of previously reported values in zebrafish (0.12–0.37; Ho *et al.*, 2002; Sun *et al.*, 2008; Lee *et al.*, 2014, 2016; Kang *et al.*, 2015; Wang *et al.*, 2017). However, the E/A ratio trended downward during acute warming, suggesting that diastolic function becomes impaired as heart rate increases with temperature (Fig. 4D).

In a notable advancement, we determined ventricular inflow volume of the ventricle using PW Doppler-derived measurements of stroke distance, together with histologically determined measures of AV valve area (Table 1). We used ventricular inflow volume as a proxy for stroke volume, assuming low variation in ejection fraction between heartbeats at a given temperature. Indeed, the ventricular inflow volumes reported here (mean of 0.43 ± 0.04 ml kg^−1^ at maximum; Table 1) are comparable to stroke volumes reported in other salmonid species (0.2–1.2 ml kg^−1^; Clark *et al.*, 2008; Steinhausen *et al.*, 2008; Eliason *et al.*, 2013; Morgenroth *et al.*, 2021). By measuring *f*_Hmax_ simultaneously with this proxy for stroke volume, this system allowed for calculations of extrapolated cardiac output across a range of experimental temperatures. Estimations of peak cardiac output made here (mean of 42.2 ml min^−1^ kg^−1^; Table 1) fall within the range of peak cardiac output values obtained in salmonids fitted with transonic blood flow probes (17–110 ml min^−1^ kg^−1^; Clark *et al.*, 2008; Steinhausen *et al.*, 2008; Eliason *et al.*, 2013; Ekström *et al.*, 2017; Morgenroth *et al.*, 2021). While these internal flow probes are commonly used to measure cardiac output in fish, test fish must be large enough for the equipment to be properly placed and supported for the duration of the experiment (Eliason *et al.*, 2013). Furthermore, presence of the instrument within the body cavity and tissue damage resulting from surgery may affect results (Clark *et al.*, 2011; Perez *et al.*, 2021). The methodology described here allows for nonsurgical estimates of cardiac output in juvenile salmonids and other small fishes.

During acute warming or aerobic exercise, cardiac output must increase to meet rising oxygen demands. However, in most teleosts, as the frequency of heart contractions increases, diastolic filling and systolic force of the heart are reduced. This creates a negative force–frequency relationship between heart rate and stroke volume, limiting a fish’s ability to increase cardiac output through increases in heart rate (Driedzic and Gesser, 1988; Shiels *et al.*, 2002). Indeed, we observed increases in *f*_Hmax_ during acute warming, concomitantly with decreases in stroke distance (Fig. 4). Interestingly, declines in stroke distance began to plateau at temperatures beyond the T_AB_ for *f*_Hmax_, when the rate at which *f*_Hmax_ increases with temperature slows. Similar patterns in heart rate and stroke volume were demonstrated in unanaesthetized rainbow trout (*Oncorhynchus mykiss*) fitted with internal blood flow probes during determinations of CT_max_ (Morgenroth *et al.*, 2021). Our findings demonstrate the utility of this system as a nonsurgical method for estimating changes in cardiac output and investigating force-frequency dynamics across a range of experimental temperatures.

The Indus DFV system was previously used to obtain heart rate and blood flow velocity measures in adult zebrafish, following cryoinjury of the heart (0.5–0.7 g; Benslimane *et al.*, 2019). Here, we extend the size range for fishes to include juvenile salmonids (10–25 g). The maximum signal range of the transducer probe sets the upper limit for specimen body size. For the 20-MHz transducer probe used here, the AV valve of the heart must be within 1 cm of the tip of the probe to obtain a proper signal. However, Indus Instruments also offers a 10-MHz transducer probe with a maximum signal range of 2 cm for use in larger animals. We also introduced a method for extending the timeframe for obtaining Doppler measurements in fish and introduced temperature control. By ram ventilating the fish in a recirculating water bath, we were able to stabilize fish in our system for up to 3 hours of experimentation and conducted measurements across a 20°C temperature range (11–31°C). However, should rapid measurements be preferred, fish need only be anaesthetized and maintained in an upside-down position for as little as 30 seconds to obtain valuable information about heart function. Recently there have been a number of studies demonstrating how thermal performance experiments can be performed on salmonids in remote field settings (Donnelly *et al.*, 2020; Gilbert *et al.*, 2020). The minimal space requirement (1 ft^2^) and handheld nature of this echocardiography system make it especially suited for use in unconventional experimental settings such as these.

The primary limitation of this DFV methodology is the requirement that specimens are immobile to allow proper placement of the probe. While there is some debate surrounding the ecological relevance of thermal tolerance estimations in anaesthetized individuals, these sub-lethal indices of thermal limitation (T_AB_ and T_Arr_) are perhaps of greater relevance to natural processes than measures of lethal thermal tolerance taken in unanaesthetized individuals (such as CT_max_), as they occur at lower temperatures that are more commonly encountered in natural settings (Gilbert *et al.*, 2020). As such, measures of cardiorespiratory thresholds for thermal tolerance are increasingly being used to predict population-specific impacts of warming river temperatures and subsequently develop evidence-based management strategies (Farrell *et al.*, 2008; Cooke *et al.*, 2012; DFO, 2012).

In summary, we showed that a mouse echocardiograph system can be used in juvenile salmon to measure key parameters of cardiac function, including *f*_Hmax_, blood flow velocity, the E/A velocity ratio and inflow volume of the ventricle. These measures can subsequently be used to approximate cardiac output and assess chamber function, painting a detailed picture of heart function. We also demonstrated how Doppler echocardiograph systems can be used to assess thermal performance of cardiac function during acute warming. Recent salmon die-offs during spawning migrations have been linked to collapses in cardiac scope due to anomalously high temperatures (Farrell *et al.*, 2008; Martin, 2019). High water temperatures are also known to limit juvenile survival, impeding conservation efforts during freshwater life stages (Crozier and Zabel, 2006). As climate change increases the frequency of record heat events and threatens wild salmonid populations, it is increasingly important to develop tools to efficiently study the cardiac physiology of these fish across life stages, as it limits thermal performance, swimming capacity and lifetime fitness.

## Author contribution

B.D.N. and S.D. jointly provided equipment and funds. B.D.N., S.D. and C.A.M. were all involved in initial ideas, experimental design, data analysis and writing of this manuscript. C.A.M. was responsible for carrying out all experiments and data collection, as well as writing the initial drafts of this manuscript.

## Funding

Natural Sciences and Engineering Research Council (NSERC) Discovery Grants (RGPIN-2017-06045 and RGPIN-2018-06665 respectively) and a joint Research Tools and Instruments grant (RTI-2018-00817) supported both B.D.N. and S.D, with additional support from a Strategic Project Grant (494220-2016 STPGP) to B.D.N. C.A.M was supported by an Ontario Graduate Scholarship, and an NSERC-Canadian Graduate Scholarship.

## Competing interests

No competing interests declared.

## Supplementary Material

CONPHYS-2021-030_Supplemental_coab070
