# Supplementary material for: Adaptation of a mouse Doppler echocardiograph system for assessing cardiac function and thermal performance in a juvenile salmonid
Source: Conserv Physiol. 2021 Sep 3;9(1):coab070. doi: 10.1093/conphys/coab070 (PMC8415535; doi:10.1093/conphys/coab070)
Supplement: CONPHYS-2021-030_Supplemental_coab070 [file conphys-2021-030_supplemental_coab070.docx]

**Supplementary materials**

**
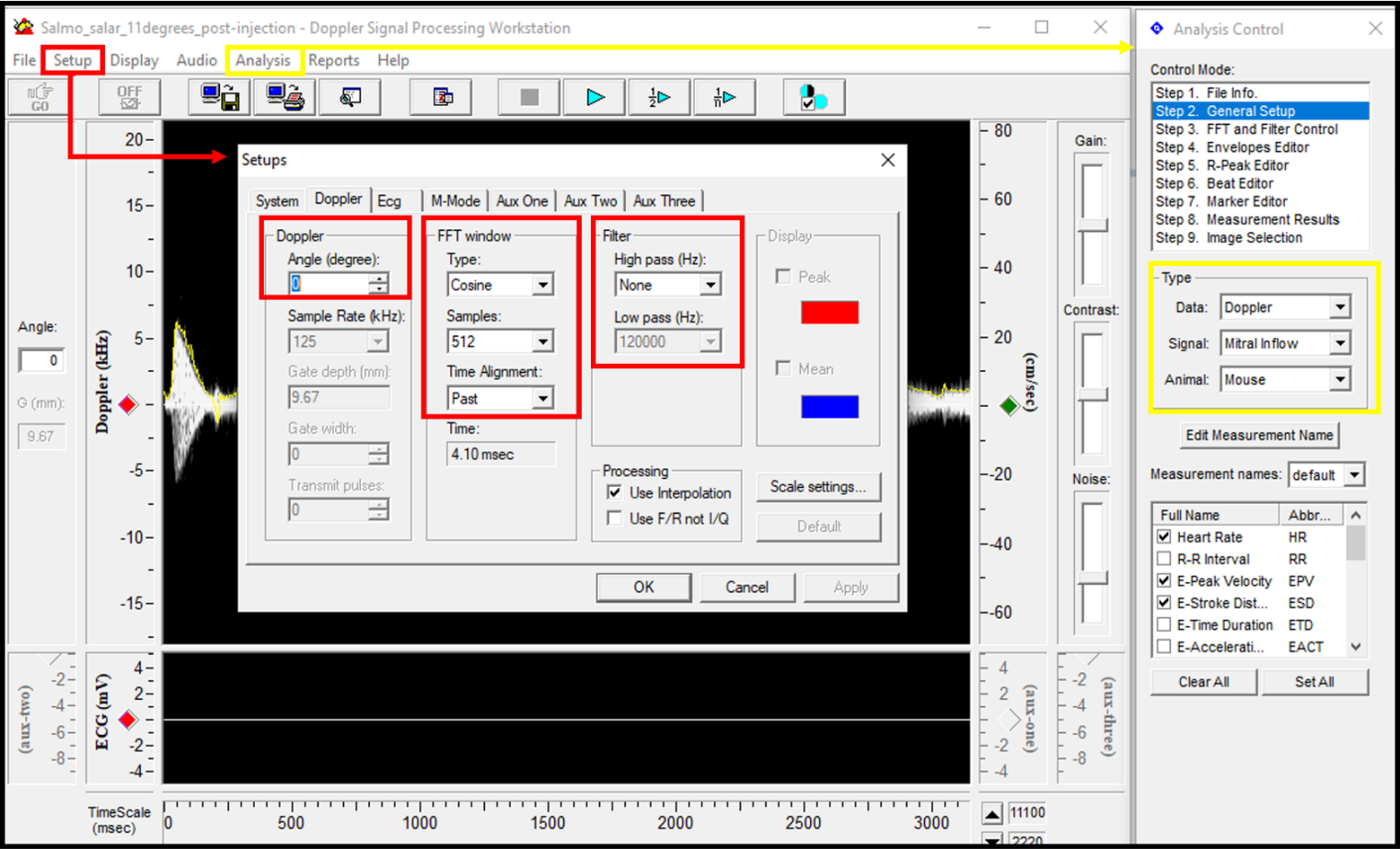
**

**Supplementary Figure 1. User interface of the Doppler Signal Processing Workstation (DSPW),** with the recommended settings for analysis of blood flow velocity at the atrioventricular (AV) valve. Prior to analysis, the angle between the probe and the direction of blood flow was set to zero under the ‘Doppler Setup’ tab in the DSPW software (Supplementary Fig. 1). Positioning the probe at a perpendicular angle, directly posterior to the gills, ensures that the direction of blood flow through the atrioventricular valve is parallel to the direction of the ultrasound beam transmitted by the probe –hence the zero-degree angle setting. Visual aspects of the spectrograph waveforms are controlled in the Fast Fourier transform (FFT) window the Doppler Setup tab. To obtain the clearest signal images, we selected ‘Cosine view; 512 samples; with past alignment’, according to the manufacturer’s instructions. Additionally, spectrograms were filtered with a low pass filter of 120,000 Hz to eliminate ambient interference. Spectrograph waveforms were then analyzed for parameters of atrioventricular blood flow using the ‘Mitral Inflow’ mode in the software’s ‘Analysis Control Window’.


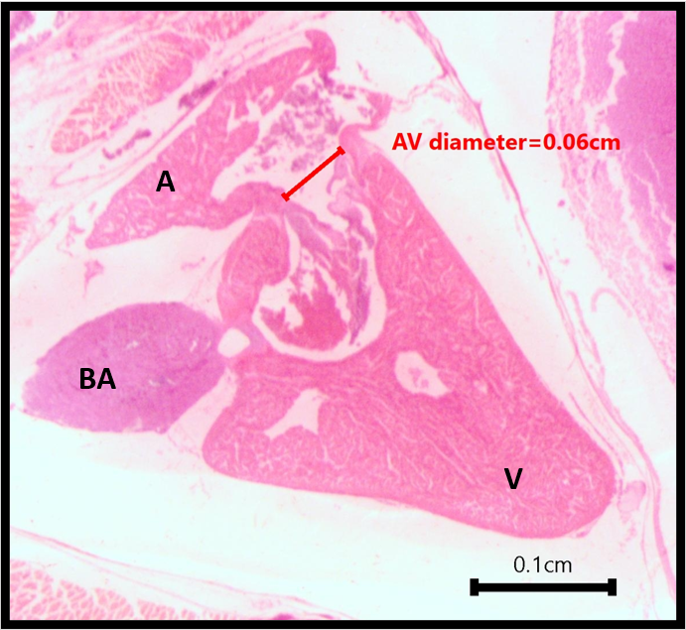


**Supplementary Figure 2. Histological determination of atrioventricular valve diameter.** Haematoxylin and Eosin (H&E) stained sagittal section of Atlantic salmon (*Salmo salar)* parr. Thickness = 10 μM; magnification = 10X; scale bar = 0.1 cm. Width of the atrioventricular (AV) valve opening (red line) was measured in OPTIKA PROView (OPTIKA Srl, Ponteranica, BG, Italy) for each slide, and the maximum diameter was then used for subsequent analysis. Abbreviations: A = atrium, BA = bulbus arteriosus, V = ventricle.
